# Managing Swedish forestry’s impact on mercury in fish: Defining the impact and mitigation measures

**DOI:** 10.1007/s13280-015-0752-7

**Published:** 2016-01-07

**Authors:** Karin Eklöf, Rolf Lidskog, Kevin Bishop

**Affiliations:** Department of Aquatic Sciences and Assessment, Swedish University of Agricultural Sciences, Box 7050, 75007 Uppsala, Sweden; Environmental Sociology Section, Örebro University, 701 82 Örebro, Sweden; Department of Earth Science, Uppsala University, 75236 Uppsala, Sweden

**Keywords:** Bioaccumulation, Boreal forest, Forestry effects, Methylation, Methylmercury, Risk governance

## Abstract

Inputs of anthropogenic mercury (Hg) to the environment have led to accumulation of Hg in terrestrial and aquatic ecosystems, contributing to fish Hg concentrations well above the European Union standards in large parts of Fennoscandia. Forestry operations have been reported to increase the concentrations and loads of Hg to surface waters by mobilizing Hg from the soil. This summary of available forestry effect studies reveals considerable variation in treatment effects on total Hg (THg) and methylmercury (MeHg) at different sites, varying from no effect up to manifold concentration increases, especially for the bioavailable MeHg fraction. Since Hg biomagnification depends on trophic structures, forestry impacts on nutrient flows will also influence the Hg in fish. From this, we conclude that recommendations for best management practices in Swedish forestry operations are appropriate from the perspective of mercury contamination. However, the complexity of defining effective policies needs to be recognized.

## Introduction

Unacceptably high mercury (Hg) concentrations in freshwater fish are observed in many regions, including Sweden. Fish Hg concentrations exceed the European Union threshold limit of 0.02 mg Hg kg^−1^ wet weight (Directive 2008/105/EC) for good chemical status in almost all of Sweden (Åkerblom et al. 2014). This is a situation that other Fennoscandian countries also face, partly due to anthropogenic contamination but also due to the background concentrations and other factors that influence Hg biomagnification, such as food web structure.

Mercury in Swedish freshwater fish originates mainly from emissions of Hg to the atmosphere that are transported long distances before being deposited in remote areas (Munthe et al. 2007b). Around half of the Hg in the atmosphere originates from anthropogenic sources such as fossil fuel combustion, metal production, cement production, waste disposal and artisanal gold mining (Pacyna et al. 2006). Forest soils are an excellent buffer for retaining Hg deposition, both from natural and more recently anthropogenic emissions (Lee et al. 2000). In the METALICUS project, Hintelmann et al. (2002) found that <1 % of the isotope marked Hg deposited in the watershed appeared in runoff within a year after deposition. Newly deposited Hg is accumulated in the organic-rich upper soil horizons where it effectively binds to reduced sulphur sites and oxygen/nitrogen-groups in the organic molecules (Ravichandran 2004; Skyllberg et al. 2006).

But even though most of the Hg deposited from the atmosphere is retained, the output from forest soils to surface waters of total Hg (THg), especially the extremely bioavailable methylmercury (MeHg) fraction is of concern. This is the starting point for much of the biomagnification of Hg in the aquatic food web that leads to unacceptably high Hg levels in fish and other biota. The main concern for forest managers is thus for the export of MeHg from forest lands. But other factors besides the actual origins of the Hg itself contribute to the Hg levels seen in fish, including mercury methylation in lakes, and the degree of biomagnification further up in the food web. The latter can be influenced by other forestry influences on aquatic ecosystems, such as nutrient release after harvest.

Measurements of MeHg in the environment reflect the net Hg methylation rate, as MeHg is simultaneously formed by methylation and degraded by demethylation. Much of the Hg methylation occurs in suboxic environments such as peatlands or lake sediments where sulphur-reducing bacteria (SRB) (Gilmour et al. 1992; King et al. 2001) or iron-reducing bacteria (IRB) (Fleming et al. 2005) among other groups are active. The Hg methylation rate is linked to factors controlling the abundance and activity of these methylators, such as the redox microenvironment, temperature and the availability of both an electron acceptor (such as sulphate) as well as an electron donor (such as high-quality organic carbon) (Drott et al. 2007). The Hg demethylation rate is suggested to be more stable than the Hg methylation rate, as the demethylation rate is influenced by both biotic and chemical factors (Skyllberg et al. 2007). The net Hg methylation rate might thereby be more influenced by factors controlling the Hg methylation rate.

**Fig. 1:**
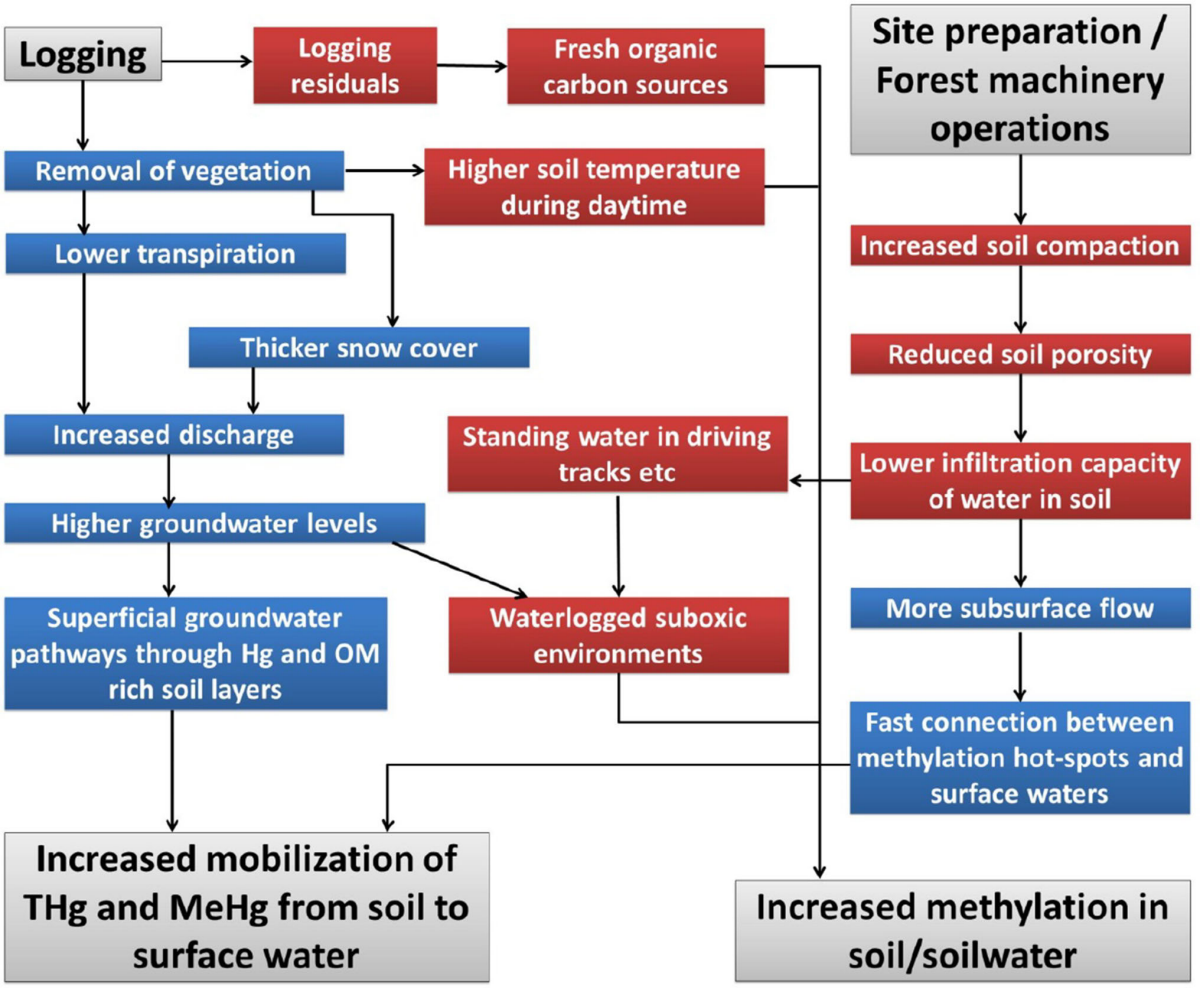
Schematic diagram of possible effects from logging, site preparation and forestry machinery driving. The effects caused by these activities are colour coded depending on whether they mainly refer to changes in (1) hydrology, including changes in soil moisture, runoff amounts, groundwater levels and groundwater flow-paths (*blue*), or (2) methylation potential, including changes in redox status, availability of electron acceptors or donators for methylation bacteria as well as soil and water temperature (*red*)

Forestry activities have been found to increase mobilization of Hg (both MeHg and the total Hg (THg) which can later be methylated) from soils to surface waters and to create environments of high Hg methylation. Forestry will also influence the aquatic food webs, and thus the degree of Hg biomagnification. The removal of trees and forestry machinery driving may influence methylation and Hg outputs to surface waters by decreasing evapotranspiration, increasing soil temperature, increasing snow cover and increasing soil compaction. The effects caused by forestry on Hg mobilization can be divided into two major groups: (1) hydrological effects, including changes in soil moisture, runoff amounts, groundwater levels as well as groundwater flow-paths, and (2) effects on the net Hg methylation rate, including changes in redox status, availability of electron acceptors or donors for methylating bacteria, and soil temperature (Fig. 1). Forestry influences on aquatic food webs include changes in nutrients, light/temperature regime and erosion.

Elevated concentrations of both THg and MeHg after forestry activities have been observed in runoff water (Porvari et al. 2003; Munthe and Hultberg 2004), downstream fishes (Garcia and Carignan 2000), zooplankton (Garcia et al. 2007) and periphyton (Desrosiers et al. 2006). Based on a review of the forestry effect studies from boreal catchments published before 2006, Bishop et al. (2009) suggested that 9–23 % of the Hg accumulated in fish in Swedish inland water was a consequence of forest harvest. However, only five published studies were available in 2006, and a number of new studies have been published since then. This review seeks to summarize the new insights from the seven relevant forestry effect studies published after 2006, with a focus on changes in fluxes and concentrations of THg and MeHg. This review categorizes the available research, in terms of effects from (1) logging, (2) site preparation and forestry machinery driving and (3) biomass removal including stump harvest and removal of logging residuals and (4) forestry activities other than the regeneration phase. We will then review recommendations for how forestry can reduce this contribution based on all the currently available literature. Since the prospects of achieving safe levels of Hg in boreal aquatic biota in the coming decade appear poor even if there was no contribution to Hg in fish from forest harvest, this paper also considers how society can address such a situation in a policy perspective.

## Effects of forestry activities

### Logging

In one of the first forestry Hg effect studies conducted in Finland, Porvari et al. (2003) identified 133 % higher MeHg concentrations in runoff after logging and site preparation than before logging. The loadings of THg and MeHg in the same study increased many-fold in the 3 years following logging (Porvari et al. 2003) as a consequence of increased discharge, commonly observed after logging. Less water will leave the area by transpiration when the vegetation is removed (Bosch and Hewlett 1982) and more snow also accumulates in open areas (Murray and Buttle 2003). Increased sunlight radiation on open ground after logging may increase soil temperatures and increase the evaporation from the soil surface, but this increase might only be of minor importance compared with the decrease in transpiration and increase in snow accumulation (Buttle and Murray 2011). Despite this, there are single-year exceptions. A study in northern Sweden found that the direct evaporation from the snow surface actually reduced the runoff during spring flood in open areas in individual years, even though the runoff was greater in other years in open areas (Schelker et al. 2013). The logging on that catchment in March also suppressed much spring flood a few weeks later due to compaction of the snow and insulation by logging slash on top of the snow (Sørensen et al. 2009a, b). Despite some spring flood exceptions, increased groundwater recharge in logged areas on the till soils of Fennoscandia generally result in more superficial lateral flow pathways that can extend up into more organic carbon- and mercury-rich superficial soils. Increased water discharge might not only increase the chemical loading of contaminants from the catchment but may also create more waterlogged, suboxic environments which provide good conditions for methylators such as SRB. Higher soil temperatures in open areas and the addition of fresh organic carbon sources from decomposition of logging residuals can further enhance the activity of the methylators (Sørensen et al. 2009a, b).

Since the alarming results from Finland (Porvari et al. 2003), several studies have been published with varying degrees of THg and MeHg response. Leaching coefficients to runoff from logged forest were 83 % higher than from growing forest for THg and 325 % higher for MeHg, when Munthe et al. (2007a) modelled the influence of the extensive forestry operations following a severe storm event in southern Sweden. The leaching coefficients were based on 4–8 streams in growing forest and 15 streams in logged or storm-felled forest. Logging followed by site preparation or stump harvest increased concentrations relative to untreated references by 22–76 % for THg and by 11–60 % for MeHg in a synoptic spatial study across Sweden (Eklöf et al. 2012). A spatial study in north-east of Sweden found a 55 % increase in THg and a 250 % increase in MeHg relative to untreated references above the marine limit (ML) for that region (Skyllberg et al. 2009). Below the ML though, there was not a significant effect of logging. Approximately 68 % of Sweden’s land area is above the marine limit. Kronberg (2014) also detected increased streamwater MeHg concentrations after logging in catchments above the ML but not below the ML. The methylation potential in logged areas was also higher than in growing forest, indicating that the increase of MeHg in soil and streamwater was mainly associated with new methylation and not just mobilization of old MeHg pools from the soil. However, not all of the newly produced MeHg reaches the stream, as the signal of the logging effects was much more pronounced for MeHg in soil then for MeHg in stream water.

If the higher concentrations of THg and MeHg in the studies that included both logging and site preparation or stump harvest were mainly due to the effects of logging or to the subsequent forestry activities is unclear. Whereas logging alone did result in significant forestry effects in some catchments (Porvari et al. 2003; Munthe et al. 2007a, b; Skyllberg et al. 2009; Eklöf et al. 2012; Kronberg 2014) no observed increases of THg or MeHg concentrations were detected after logging in some other catchments (Allan et al. 2009; Sørensen et al. 2009a, b; de Wit et al. 2014; Eklöf et al. 2014). In another Swedish study (Balsjö in north-east of Sweden), the loads to surface waters did increase for both THg and MeHg after logging, even though the concentrations did not change significantly (Sørensen et al. 2009a, b; Eklöf et al. 2014).

Not only Hg but also the runoff of other solutes can be influenced by logging. This in turn could influence the Hg biogeochemistry and the Hg bioaccumulation in the food web. A number of studies have found increased dissolved organic carbon (DOC) and nitrate concentrations as well as loads after logging (Kreutzweiser et al. 2008; Schelker et al. 2012). As Hg and other trace metals bind to organic molecules, an increase of DOC can also result in increased mobilization of Hg. However, not only the quantity of DOC, but also the quality of DOC can change as a consequence of logging. O´Driscoll et al. (2006) suggested that more superficial flow paths after logging and a mobilization of less degraded organic molecules that may bind more Hg will make the Hg less available for photo reduction that can promote the production of dissolved gaseous Hg (DGM) in the water phase. The production of DGM is one of the processes that removes Hg from the water phase by volatilization. The effect of changed dissolved organic matter (DOM) quality after logging may thereby result in more Hg staying in the water column, in addition to other effects on the aquatic ecosystem. Higher nutrient loadings after harvest may influence Hg bioaccumulation. de Wit et al. (2014) found a decrease in MeHg levels in herbivorous stoneflies after harvest, possibly as a result of higher nutrient loadings and thereby higher diet availability in the stream from a harvested catchment. Measurements of dietary biomarkers (δ^15^N signature) in the stoneflies supported higher diet availability after harvest.

### Site preparation and forestry machinery driving

Site preparation refers to the deliberate disturbance of the soils prior to the planting of new trees. This mechanical treatment exposes the mineral soil and forms mounds or ridges where the new seeds or seedlings will have a better chance of surviving. Forestry machinery operations during logging, site preparation and stump harvest, can affect the soil physical properties, the hydrological regimes and the erosion rate (Cambi et al. 2015). Significant increases in soil compaction are commonly observed after forestry operations but the magnitude of the disturbance caused by compaction varies with factors such as climate, soil properties and management practice (Greacen and Sands 1980). The reduction of soil porosity might lower the infiltration capacity of water in the soil (Kozlowski 1999). This could increase the superficial flow and result in flooded soils in logging tracks and other local depressions. Flooded soils can act as Hg methylation hot-spots, with low redox potentials and good access to fresh organic carbon sources (Porvari and Verta 1995; Hall et al. 2005). Overland flow, that connects methylation hot-spots to surface waters, could then increase the load of MeHg to aquatic ecosystems (Bishop et al. 2009). Increased erosion has also been found to be a consequence of forestry machinery operations (Kozlowski 1999), which could also lead to increases in Hg loads, as the eroded particles and associated Hg are exported to streams and water bodies.

A severe forestry effect on MeHg was documented in south-west Sweden where forestry machinery driving disturbed the soil when passing a stream channel (Munthe and Hultberg 2004). The MeHg concentrations downstream of this disturbance increased by 460 % and the increase has persisted for many years. No forestry effect was caused by logging on the concentrations of THg and MeHg in runoff from the Balsjö catchments in north-east Sweden, but concentrations increased by around 30 % for THg and 50 % for MeHg after site preparation compared to the situation before logging (Eklöf et al. 2014). The study of Munthe and Hultberg (2004), and the findings in Eklöf et al. (2014), indicate that not only logging operations but also soil disturbance from forestry machinery could result in significant forestry effects on Hg. Munthe and Hultberg (2004) suggested that increased MeHg concentrations were a consequence of changed water flow pathways that mobilized MeHg from the soil pool.

### Forest biomass harvesting: Stump harvest and logging residual removal

Forest biomass harvest refers to the harvest of additional tree biomass besides the stems used for forest products, e.g. stumps and logging residuals. Stump harvest is the extraction of the stumps to maximize the supply of biofuels from the harvest. Removal of stumps might disrupt the physical structure of the soil, but the magnitude of the soil disruption depends on the architecture of the roots (Walmsley and Godbold 2010). Forestry machinery operations on soils where the roots are extracted may thereby cause more soil compaction. Furthermore, more extensive operation of forestry machinery during stump harvest compared with conventional site preparation could result in even more compaction and disturbance of the soils. The stump harvest is often followed by traditional site preparation as well, although the stump harvest itself suffices as soil preparation in some sites. Although there is a higher risk of more severe soil disturbance during stump harvest compared with conventional site preparation, two studies in Sweden comparing stump harvest with site preparation have found no differences in the THg or MeHg concentrations of stream runoff (Eklöf et al. 2012, 2013). An investigation of biomass harvesting by Mitchell (2012) detected no further increase in areas of biomass removal (around 85 % slash removal) compared to conventional logging operations in terms of runoff Hg concentrations. Unpublished data from Sweden suggest that stump harvest can cause a higher frequency of methylation hot-spots (i.e. areas of high Hg methylation) compared to conventional site preparation. The elevated MeHg concentrations in these hot-spots, however, did not result in any signal in the runoff water in the studied catchments (Eklöf et al. unpubl.).

Increased amounts of forest biomass removal could also be achieved through higher logging intensity with shortened rotation periods. Removal of logging residues that is the most common way to increase the amount of forest biomass after ordinary logging, may decrease shading and increase water losses through evaporation. Logging residues, used to protect the soil from driving damages during forwarder traffic, have been found to increase the methylation in groundwater directly under the logging roads (Eklöf el al. unpubl.). This might be an effect of additional high-quality carbon sources for Hg methylators. A removal of logging residues could thereby decrease the Hg methylation in the area, however, due to the complex influences mentioned above that can work in different directions, it is difficult to predict the overall tendency in runoff Hg concentrations created by increased biomass removal through harvesting stumps and slash.

In summary, more research is needed to reveal how forest biomass harvesting operations influence THg and MeHg runoff, especially on the long-term effect of biomass removal on THg and MeHg in runoff. It is also important to learn more about how intensified forestry effects hydrology and soil chemistry, including changes in nutrient status and carbon stocks as well as soil structure, since these are factors that may have long-term importance for Hg methylation and mobilization.

### Forestry activities others then the regeneration phase

There is a general dearth of information about the influence of most forestry activities on mercury outside the regeneration phase (i.e. harvest and site preparation). To our knowledge, there are no studies on the effects of Hg mobilization and methylation as a consequence of thinning, soil fertilization, ash return or even forest drainage. There is, however, one recent study that examined the effect of ditch cleaning. A large pulse of Hg and MeHg was observed during the first days after the ditch cleaning, but this subsided after a few days (Hansen et al. 2013).

## Mercury in freshwater fish as a consequence of forestry activities at a landscape scale

Bishop et al. (2009) estimated how much of the mercury in Swedish freshwater fish could be attributed to forestry. To do this, they made three major assumptions: (1) one percent of the landscape is harvested each year; (2) the harvest impact will persist for a decade; and (3) the concentration of mercury in runoff leaving these harvest-impacted areas is two to four times that leaving established forests. Bishop et al. (2009) pointed out that there was a large amount of uncertainty surrounding that last assumption given how few studies had been published and the large variation in those reported forestry effects. The sites that had been studied might also not have been representative at a landscape scale, since large regional variations may occur. The knowledge about the release of MeHg from managed growing forest was also poorly defined.

Since 2009, some new insights into those assumptions have come to light: Bishop et al. (2009) estimated their impact from forestry on the basis of increases in the load of MeHg, not concentration. But, the MeHg concentrations in water have been found to correlate with MeHg in biota in several studies, especially at the base of the food chain (Paterson et al. 1998). This suggests that it is the concentration of MeHg in water that is of importance, not the load of MeHg. The load may still be of importance though. If both the load and the concentrations from a tributary in a lake catchment increase, then a higher fraction of the water with high MeHg concentrations will reach the lake. Although we suggest the concentrations of MeHg to be more important than the fluxes, there is most probably not a linear relation between MeHg concentrations in the water and MeHg in biota higher up in the food chain. Especially not as forest management, beyond the effects on the cycling of Hg itself, influence the structure of aquatic ecosystems. Some, but by no means all, of these influences are associated with increases in nutrient loadings after harvest, as well as erosion, light and temperature regime changes (Kreutzweiser et al. 2008). These forestry influences manifest themselves in aquatic ecosystems in a variety of ways, which can influence the degree of bioaccumulation (Lucotte et al. 2012).

Another uncertainty is scaling across stream networks. Most of the available studies have been conducted in small catchments. Such headwaters usually have a high proportion of solutes deriving from the terrestrial areas, and in-stream processes are less important, compared to in higher order streams or lakes further downstream, where in-stream processes such as photodemethylation are important for the MeHg budget (Poste et al. 2015). Low-order streams are also not the habitats where most fish biomass is found. The fate of MeHg delivered to headwaters as a consequence of forestry is unclear as it moves downstream to higher stream orders. The effect of downstream transport through the stream network on MeHg has not been defined. However, in-stream processes increase in importance for many other biogeochemical processes, such as organic carbon transformations, the further downstream one moves in the stream system (Webster and Meyer 1997). Terrestrially derived MeHg might be transformed to inorganic Hg as water moves downstream. The relative importance of terrestrially derived MeHg will then be of less importance the further down in stream order one moves. Terrestrially derived MeHg can be photodegraded, and new sources of MeHg exist in lake sediments where methylation can occur. This can further attenuate the influence of forest harvest. There is thus a question about the extent to which the “forestry signal” of increased MeHg concentrations in headwaters is manifested in the Hg concentration of fish that are mainly found further downstream in high-order streams or lakes. Another important factor is that the fishes are often at the top of the food chain in aquatic ecosystems. The organisms at the base of the food chain are also present in the low-order stream systems receiving the direct inputs of runoff from forestry-impacted catchments. Although the turn over time of MeHg may be fast, MeHg accumulated in phytoplankton or zooplankton may be transported for long distances and biomagnified in higher trophic levels when moving downstream. However, such scaling phenomena have yet to be addressed for MeHg.

The location of the forestry operations in the catchment is also likely to affect the contribution of forestry to elevated MeHg in water and biota. All the available studies are from sites where the treated area extends to the stream edge (though in some cases with a 10 metre riparian buffer zone). If the treated area is situated in the upper parts of the catchment with more intact forest between the harvest and the stream, the forestry impact may also be reduced.

Despite the uncertainties in the Bishop et al. (2009) estimate of forestry contributions to Hg in fish, it remains one of the few such estimates. In this review, we will use the calculations in Bishop et al. (2009) as the starting point for considering what more recent studies can tell us about forestry’s influence on mercury in boreal fish in terms of Hg exports from the forest landscape.

### Large variation in forestry effects among sites

There is a great deal of variation in the treatment effects on THg and MeHg among different sites. Studies with or without significant forestry effects are summarized in Table 1. No observed increases of MeHg concentrations were detected after logging on boreal catchment study sites in Ontario in Canada (Allan et al. 2009), in southern Norway (de Wit et al. 2014) and after logging only (before site preparation) in north-east of Sweden (Sørensen et al. 2009a, b). Significant increases of MeHg of less than 76 % were observed in north-east of Sweden (Eklöf et al. 2014; Kronberg 2014) and in a spatial study over all of Sweden (Eklöf et al. 2012). Significant increases in MeHg of more than 100 % up to 325 % were detected in Finland (Porvari et al. 2003), southern Sweden (Munthe et al. 2007a) and north-eastern Sweden (Skyllberg et al. 2009). The study of Munthe and Hultberg (2004) demonstrated the significance of driving damages in connection to surface waters that increased the MeHg concentrations by 460 %, but this study did not include a traditional harvest.

**Table 1:** Significant treatment effects in surface water, caused by different kinds of forestry activities, in various forestry impact studies in boreal and hemiboreal catchments in Scandinavia and North America

Publication	Treatment	Region	Increase of (THg)?	Increase of (MeHg)?	Comment
Kronberg (2014)	Logging	North-east Sweden	–	40–60 %	Calculated increase of MeHg in logged areas with undulating topography (60 %) and catchments with flatter land (40 %), based on MeHg export data from clear-cuts, growing forest and wetlands in Sweden
Eklöf et al. (2014)	Logging	Balsjö, north of Sweden	No	No	Increased load of THg and MeHg (30–50 %).
Eklöf et al. (2014)	Site preparation	Balsjö, north of Sweden	30 %	50 %	Larger treatment effect on concentrations from site preparation than antecedent logging
de Wit et al. (2014)	Logging	Norge	No	No	No forestry effect although intense soil disturbance caused by logging
Eklöf et al. (2013)	Stump harvest	Örebro, Sweden	No	No	No treatment effects caused by stump harvest, but logged areas in general higher than references. However, the study did not include logging effects
Eklöf et al. (2012)	Logging and Stump harvest or Site preparation	North, middle and south of Sweden	11–60 %	22–76 %	Stump harvested and site prepared areas significantly higher than references, but no difference between stump harvest and site preparation
Skyllberg et al. (2009)	Logging and site preparation	North Sweden	55 %	250 %	Significant increase of MeHg only in areas over highest coastline
Munthe et al. (2007a)	Logging	South Sweden	83 %	325 %	The numbers stated here are the numbers that the authors used as leaching coefficients for logged forest contra growing forest, based on measurements in 4-14 logged or unlogged catchments in south Sweden
Sørensen et al. (2009a, b)	Logging	Balsjö, north of Sweden	15 %	No	Increased load of THg (20–30 %) due to increased discharge.
Allan et al. (2009)	Logging	Canada	No	No	No increase of THg and MeHg detected in stream water, but in some areas forestry caused increases in soil- and ground-water
Munthe and Hultberg (2004)	Driving track	Gårdsjön, Sweden	31 %	460 %	Driving track crossing a former reference stream
Porvari et al. (2003)	Logging and site preparation	Finland	48 %	133 %	Loads of THg and MeHg increased up to a factor of 10

Bishop et al. (2009) estimated that 9–23 % of Hg in fish is a consequence of final felling, based on the consensus reached during an international symposium about forestry effects on water and biota. In that earlier estimate, the assumption that 1 % of the landscape is impacted each year and the impact will remain for 10 years has not been contradicted by new studies. Therefore, at any given point in time 10 % of the landscape may be impacted and 90 % unimpacted. The main change as a result of new studies is that there is a possibility for greater variability in forestry response, including little or no effect on THg and MeHg concentrations. Consequently, the changes at individual sites can be outside the range of 9–23 % in Bishop et al. (2009). Since new studies include some examples of very low impacts of forestry on the concentration of THg and MeHg change after harvest, this indicates that the mean effect of forestry will be somewhat lower in terms of the total amount of THg and MeHg released. But it is clear that there is a significant detectable forestry influence on Hg after forestry activities in synoptic, landscape scale studies. The forestry influence cannot be discounted. There is also greater recognition that bioaccumulation of Hg is related to more factors than just the amount and form of Hg in water. Since forestry influences many aspects of aquatic ecosystems, this will also influence bioaccumulation, though the degree and even the direction remain difficult to predict.

## How can society handle the problem of Hg in the environment?

Based on the above analysis, the challenge facing society today is extremely complex. The ecological situation is that most lakes in Sweden have a concentration of mercury in the biota that exceeds the European guidelines for good ecological status (Directive 2008/105/EC). The primary cause of this is industrial activities, not least fossil energy. Forest soils retain much of the anthropogenic mercury deposited from the atmosphere. Forest operations, however, mobilize mercury, resulting in increased biomagnification in aquatic ecosystems. Forestry will also influence the food web structure, and thus the pathways of bioaccumulation. Simultaneously, increased bioenergy from forests can reduce consumption of fossil fuels. Thus, long-term strategies for decreasing emissions of mercury (through intensified forestry) may lead to increased leakage of mercury, due to the soil disturbance from forestry. The issue is further complicated by the considerable variation in forestry effects at different sites. It is also uncertain to make calculations about the contribution of forestry to Hg in the biota since there are many ways in which the food web and bioaccumulation are altered by forestry. Also, even if forestry operations could be managed to make no contribution to Hg mobilization, this will not alone solve the general problem of mercury in aquatic ecosystems. Thus, while the problem of mercury in the forest landscape is a challenge that needs to be handled, at the same time it is an extremely complex task to both allocate responsibility as well as develop relevant and viable countermeasures.

From a governance perspective, the task is to render the issue of mercury in environments manageable. Research has shown that it is often difficult to find solutions when knowledge is uncertain, when the issue at stake is prioritized differently by the involved actors and when they even have different conceptualizations of the problem (Lidskog et al. 2011). If no general definition is agreed upon, then there is rarely any opportunity to formulate a joint plan for concerted action (Palmer 2012).

In response to the situation of increasingly complex environmental issues, the notion of ‘risk governance’ has been developed (van Asselt and Renn 2011; Lofstedt et al. 2011). Due to the complexity of the many problems it is important that experts involved are open to question the situation; that issues of uncertainty are not concealed and that regulators are receptive to the input and participation of stakeholders. Research has also shown that in order to shape regulatory arrangements, it is important to reduce the complexity, create a spatial identity and allocate responsibility for the issue at stake (Lidskog et al. 2011). It is not within the scope of this paper to discuss options for dealing with the problem of mercury export from Sweden’s managed forest landscape in detail, but we do try to provide some thoughts about the direction for future work.

### Uncertainty

To make complex phenomena governable, complexity must be reduced and uncertainties need to be managed. For this issue, there are a large amount of uncertainties when calculating forestry effects with regard to mercury bioaccumulation and to what extent findings in one catchment are valid for another catchment. According to the high variation in the forest effect studies presented here, the influence of a treatment may differ dramatically depending on where a harvest is located relative to the stream network, as well as soil structure, chemistry, topography and wetness. However, it is important to note that this scientific uncertainty may not necessarily constitute a hindrance for developing policies. A common way to manage uncertainties is to acknowledge them, making them transparent for non-scientific actors and open up a space for discussing what should be seen as acceptable risk and costs. In this case, there is a need to take decisions with explicit reference to non-scientific fundamental principles and values. By drawing boundaries for what is acceptable and developing systems for controlling risk, even issues attached with great uncertainty can be made manageable.

### Responsibility

Many forest operations seem to lead to increased leakages of mercury to aquatic environments. Mercury is not originally delivered by forestry itself, but as airborne emissions from other human activities as well as natural sources. However, forestry has a responsibility to consider its negative impacts on the forest’s capacity to buffer and mitigate the pollution created by other sectors in other parts of the world. At the same time, it is not realistic to claim that forestry alone should take responsibility since the forest only functions as a buffer against the pollution created by other activities. As for many other environmental issues there is a need for all actors that are part of the cause of a problem to take responsibility. For forestry it means to consider how it is possible to minimize environmental consequences without losing the other important ecosystem services that forests provide. This leads to the issue of scaling; the importance of finding appropriate spatial and temporal perspectives.

### Scaling

In order to develop relevant regulatory arrangements, there is a need to decide appropriate temporal and spatial boundaries. It is important to not only stress mercury leakages from the forest harvest but to consider the whole forestry cycle’s contribution to mercury exports and bioaccumulation in aquatic ecosystems, including changes in food webs. Also, it is important to consider the positive long-term effects of forest management for reducing mercury emission (not least by substituting fossil energy), and by reducing waterlogged hot-spots for Hg methylation. Focus should not just be on the negative impacts in terms of increased leakages to the aquatic environment during the harvest phase of forest management. Furthermore, it is important to not one-sidedly focus on the contribution from forestry. Putting the mercury export from Swedish managed forest landscapes in a broader spatial and temporal perspective helps to avoid sub-optimal regulations.

For mercury, the current challenge is to find how much responsibility forestry should take for minimizing its contribution to the bioaccumulation of mercury in the environment. As shown in this paper, forestry cannot avoid responsibility, but should share it with other actors. This is due in part to forestry not being the primary source of the Hg pollution, but also due to the importance of not threatening ecosystem services provided by forestry.

## Recommendations for forestry practice

The Swedish Forest Agency (2014) is responsible for seeing that forest owners take suitable precautions to protect the natural environment when conducting forestry operations. In the spirit of the precautionary principle, these activities should not degrade water quality. The actual standards for water quality criteria that have to be reached, including the impacts from forestry activities, are set by Sweden’s five water districts in accordance with the European Union Water Framework Directive. The Swedish Forest Agency has formulated a set of guidelines, some of which are mandatory to follow and some of which are just recommendations. These guidelines aim to protect water quality in general, and in this way they all are relevant to reducing the human influence on aquatic food webs that structure the way mercury moves of the food chain. Some of the guidelines are also appropriate to preventing increased mobilization of THg and MeHg to surface waters. They are as follows:

- Avoid creating large contiguous harvest areas, especially if the areas are wet or the soils are fine grained.
- Minimize negative impacts on water environments by co-operating with neighbouring land owners to avoid direct connection to lakes, streams and wetlands, as well as locating forest roads on drier parts of the landscape.
- Forestry machinery driving should be done in such a manner that mobilization of particles is minimized, water flow pathways do not change, and no impoundments or wet areas are formed along streams. Peat-land surrounding streams and lakes should not be damaged. Driving on wet areas should be avoided and it is recommended to protect the forest floor by using logging residuals or logging mats when passing wet areas or water courses.
- Buffer zones with trees and vegetation should be left to such an extent that they prevent negative effects on surface water quality.
- Site preparation should not be conducted in buffer zones along lakes, streams or wetlands. Site preparation should be conducted in such a manner that erosion is avoided.
- Stump harvest should not be conducted in buffer zones, in wet areas or on steeply sloping terrain. It is recommended to avoid stump harvesting on fine-grained soils.
- When constructing new ditches or cleaning old ones, these should stop before they reach streams or lakes unless actions are taken to prevent increased mobilization of particles. Humus traps could be used to prevent the mobilization of particles downstream.

All the Swedish Forest Agency guidelines above are consistent with the knowledge gained from available forestry effect studies. By avoiding driving, site preparation and stump harvest in wet areas and using soil protection when passing wet areas, the formation of Hg methylation hot-spots can be reduced. Buffer zones along streams, wetlands and lakes also reduce hydrological connections between surface waters and possible hot-spots or areas of soil erosion in the treated areas.

On the basis of the available forestry effect studies there are several Swedish Forest Agency recommendations that merit extra attention as they appear particularly important for preventing Hg mobilization and methylation:

- Avoid hydrological connections between methylation hot-spots and surface waters.
- Take weather conditions into account when planning and conducting forestry activities. Logging on snow cover and soil frost are preferred. Avoid forestry activities after a storm when the areas are very wet.
- Take the local topography, wetness index and carrying capacity into account when planning where to do a certain forestry activity, where to drive with forestry machinery and where to locate forest buffers.

Different kinds of forestry activities can form methylation hot-spots. However, the signal from these hot-spots does not always appear to be that strong in the runoff water (Eklöf et al. unpubl.; Kronberg 2014). If the hydrological connection between hot-spots and surface waters can be minimized, the MeHg formed in the hot-spots in treated areas has less chance to reach surface waters before being demethylated. Buffer zones along streams, wetlands and lakes are to prevent fast connections between methylation hot-spots and surface waters.

One other guideline that deserves special attention is when to do forestry. The study in Balsjö in north-east of Sweden (Sørensen et al. 2009a, b; Eklöf et al. 2014) suggested that the lack of logging effect on the THg and MeHg concentrations could be a result of the minimal soil disturbance during winter harvesting conditions when snow covered the ground. Not all forestry operations can be conducted during winter conditions, but logging, stump harvest and forestry machinery driving should preferably be avoided when the soil is wet after a storm event or a long rain period.

Buffer zones are already one of the prioritized guidelines from the Swedish Forest Agency, but these guidelines would benefit from refinement. Kuglerová et al. (2014) suggested that the width of buffer zones should vary depending on site-specific characteristics, not only between sites but also along a specific stream. Wider buffer zones are needed in groundwater discharge areas where the hydrological outputs are concentrated and driving damage sensitivity is high. Narrower buffer zones could be allowed in areas with less groundwater discharge and less ecological significance (Kuglerová et al. 2014). Such site-specific precautions should preferably also be used when planning the location of operations such as logging, stump harvest and the driving of forestry machinery.

## Conclusions

This review underlines the challenge of dealing with the environmental problem of unacceptably high levels of Hg in freshwater fish in general, and the role of forestry in particular. Although there are now more studies on the effects of forestry on MeHg in surface waters, the magnitude of that forestry effect is still uncertain. This is partly due to the high variation between different forestry effect studies, and partly due to the fact that forestry’s influence on mercury in fish goes beyond just mobilizing mercury to altering the aquatic foodwebs that structure the bioaccumulation of Hg which eventually reaches fish. The earlier estimate of 9–23 % of Hg in Swedish freshwater fishes arising from forestry (Bishop et al. 2009) may be too narrow and high, missing some sites that have recently been reported where there is a low degree of influence after forest harvest operations. Most available forestry effect studies focus on MeHg runoff in low-order streams. Calculating Hg at the top of the food chain adds layers of uncertainty associated with bioaccumulation, Hg transformation in downstream systems and the importance of in-lake processes, all of which forestry influences in ways beyond the actual mobilization of mercury. A variation in forestry effects may also arise from the location of forestry activities within a catchment and catchment-specific properties such as topography, wetness and the chemical properties of different catchment soils. Despite the uncertainties, however, this review shows that there are measurable effects of forestry in the regeneration phase, and the possibility remains for larger contributions in specific areas. Most available forestry effect studies also focus on the regeneration phase (i.e. logging and site preparation), and we stress the importance of focusing on the entire forestry cycle over the course of 50–100 years when trying to assess forestry’s overall effects. Finally, there is another set of issues beyond simply apportioning a fraction of the MeHg to fish; how should the forestry sector address this fraction in a way that accounts for both the gravity of mercury as an environmental pollutant, and other ecosystem services provided by forestry? We suggest the value of creating spaces for discussing and deliberating viable measures and trade-offs for governing this complex issue. This is a discussion that should be conducted both within the forest sector but also on a general societal level since this issue cannot and should not be addressed by forestry alone.
